# Polymer Inclusion Membranes

**DOI:** 10.3390/membranes12020226

**Published:** 2022-02-16

**Authors:** Eduardo Rodríguez de San Miguel

**Affiliations:** Departamento de Química Analítica, Facultad de Química, Universidad Nacional Autónoma de México (UNAM), Ciudad Universitaria, Ciudad de Mexico 04510, Mexico; erdsmg@unam.mx

Polymer inclusion membranes (PIMs) are a kind of membrane in which a carrier is physically trapped within a polymer network usually in, but not restricted to, the presence of a plasticizer. Due to their specific advantages such as easy synthesis, effective carrier immobilization, versatility, and good mechanical properties, they are thought to be a great alternative to supported liquid membranes (SLMs), where a liquid phase containing the carrier and the solvent are impregnated within the pores of a polymeric film. Although there are some similarities between both membrane types, the physical nature of the PIMs easily allows their integration within different chemical systems making them suitable for diverse applications in addition to the well-established extraction and separation processes of metal ions and small organic molecules, e.g., optode and catalyzer development, energy conversion and passive sampling, speciation measurement, sample pretreatment, and metal nanoparticle synthesis. However, despite such potentiality, there are still many important issues that are worth being studied concerning PIMs, as denoted by the variety of themes recently addressed in the Special Issue in *Membranes* dedicated to them, “Polymer Inclusion Membranes”. 

In this regard, concerning PIM transport modeling, P. Szczepański [1] proposed a new model to describe the transport kinetics. The new model, based on an equation similar to the first-order chemical reaction equation with equilibrium, was successfully applied to the simultaneous transport of Zn(II), Cd(II), Cu(II), and Pb(II) with di-(2-ethylhexyl)phosphoric acid (D2EHPA) as a carrier, o-nitrophenyl octyl ether (NPOE) as a plasticizer, and cellulose triacetate (CTA) as a polymer matrix. The results indicated that the calculated initial fluxes (from 2 × 10^−11^ up to 9 × 10^−10^ mol/cm^2^·s) were like the values observed by other authors in systems operating under similar conditions. The results also showed that the application of a model based only on an equation similar to the first-order chemical reaction, as performed in many studies, is severely limited. In some cases, because of abnormally distributed residuals, the assumption of linearity does not hold properly. This may lead to an underestimation of permeability coefficients and initial maximum fluxes. Only for systems in which the transported substance reacts immediately in the stripping solution, creating a product which is not transported through the membrane, such simplified approach seems to be justified. The proposed model, in addition to being more flexible, provided the best nonlinear fit to the experimental data. As the author mentioned, the fact that although an increase in difficulty for its application is related to the simultaneous estimation of two parameters, actual computer software provides a very simple way to overcome such circumstance.

On the other hand, concerning PIM synthesis, recently deep eutectic solvents (DESs) emerged as a new generation of Ionic liquids (ILs) prepared by mixing a hydrogen bond acceptor (HBA) and a hydrogen bond donor (HBD) with remarkably low volatility. As such, they are suitable to be used in extraction processes. DESs may also play the role of transport carrier for amino acids because of their amphoteric nature. Thus, Matsumoto et al. [2] studied the permeation of lactic acid from the cultures through a poly(vinyl chloride)(PVC)-based PIM that contains DESs as a carrier as a simple and environmentally benign technique for lactic acid separation on an industrial scale. Lactic acid was successfully permeated through PIMs containing hydrophilic DESs, urea-choline chloride and glucose-choline chloride. Hydrophobic DESs were unsuitable as a membrane carrier for PIMs because of a low permeation rate. Simple preparation of thinner membranes in the PIM process and higher permeation rates were advantages over the SLM process. The permeation behavior was explained by the facilitated transport mechanism based on the solution-diffusion model.

As for the structural characterization of PIMs, Mancilla-Rico et al. [3] characterize membranes containing cellulose triacetate as support, Ionquest^®^ 801 ((2-ethylhexyl acid) -mono (2-ethylhexyl) phosphonic ester) as extractant, and 2NPOE (o-nitrophenyl octyl ether) or TBEP (tri (2-butoxyethyl phosphate)) as plasticizers using several instrumental techniques (Fourier Transform Infrared Spectroscopy (FT-IR), Reflection Infrared Mapping Microscopy (RIMM), Electrochemical Impedance Spectroscopy (EIS), Differential Scanning Calorimetry (DSC)) with the aim of determining physical and chemical parameters (structure, electric resistance, dielectric constant, thickness, components’ distributions, glass transition temperature, stability) that allow a better comprehension of the role that the plasticizer plays in PIMs designed for In(III) transport. The results showed that in comparison to TBEP, 2NPOE presented less dispersion and affinity in the PIMs, a plasticizer effect at higher content, higher membrane resistance (R_mem_) and less membrane permittivity (ε_r,m_), and a pronounced drop in the glass transition temperature (T_g_) values. However, as In(III) was absorbed by the PIM, these parameters changed, and an increase in ε_r,m_ and a decrease in R_mem_ were observed, this effect being more pronounced for 2NPOE than for TBEP. RIMM analyses showed that the distribution of 2NPOE on the polymeric support was homogeneous; for TBEP and Ionquest^®^ 801, irregular distributions were obtained, and a lower presence of these organophosphate components with respect to those with 2NPOE. However, when ternary systems were formed, the distributions changed. TBEP showed more affinity for Ionquest^®^ 801 than 2NPOE, and the compatibility of the extractant and plasticizer increased with the augment in plasticizer content for both systems. Composition regions were additionally established for the synthesis of the membranes in a wide range. The areas for non-favorable PIM formation were for 2NPOE and TBEP, respectively: CTA < 15%, Ionquest^®^ 801 > 40% and 2NPOE < 50%, and CTA < 15%, Ionquest^®^ 801 > 55% and TBEP < 35% mole/mole. PIMs with TBEP accepted more Ionquest^®^ 801 and required less plasticizer content in comparison to those with 2NPOE for a positive formation, indicating a better affinity of Ionquest^®^ 801 for TBEP than for 2NPOE. PIM thickness measurements supported this statement. In conjunction all the information suggested a better plasticization efficiency of NPOE, which seems to be phase-separated, that in the presence of the cation gave rise to a medium of high mobility and polarity, where the structural change promoted by the plasticizer was a key factor in the transport efficiency of the PIM system. A drawback was the decrease in stability because of the minor affinity among the components in 2NPOE-PIMs.

Finally, new forms to evaluate the chemical information provided by PIM optodes were analyzed by García-Beleño and Rodríguez de San Miguel [4] through an optimization of the composition of PIM-based optodes, and their exposure times to metal ion solutions (Hg(II), Cd(II), and Pb(II)) using two different chromophores, diphenylthiocarbazone (dithizone) and 1-(2-pyridylazo)-2-naphthol (PAN), using a chemometric approach. In their work, Derringer’s desirability functions values were employed as response variables to perform the optimization obtained from the results of three different processes of spectral data treatment: two full-spectrum methods (M1 and M3) and one band-based method (M2). The three different methods were compared using a heatmap of the coefficients and dendrograms of the Principal Component Analysis (PCA) reductions of their desirability functions. The final recommended M3 processing method, i.e., using the scores values of the first two principal components in PCA after subtraction of the normalized spectra of the membranes before and after complexation, gave more discernable differences between the PIMs in the Design of Experiments (DoE), as the nodes among samples appeared at longer distances and were varyingly distributed in the dendrogram analysis. As M3 focuses on the relevant changes after the complexation of the chromophore and the metal has occurred, the developed full-spectrum method can be used when band-based methods present problems related to overlapping, shifting, and distortion of the signals. In addition, it does not suffer drawbacks associated with the interpretability of full-spectrum methods based only on PCA. Due to its easy chemical meaning and the adequate determined color changes, the method was recommended as a novel optimization method for this kind of PIM optode. Applications to multicomponent detection to deconvolute a more complicated system with even more metal ion components were promising areas of future research highlighted by the authors.

In conclusion, it is expected that new findings and contributions in the field of PIMs will be continuously appearing in the literature, as new areas for their applications, modification in their synthesis, structural characterization, theoretical modeling, and the analysis of the obtained chemical information emerge. This Special Issue provides readers with some highlights of such developments.

## Data Availability

Not applicable.

## References

[1] Szczepański P. (2020). Some Critical Remarks about Mathematical Model Used for the Description of Transport Kinetics in Polymer Inclusion Membrane Systems. Membranes.

[2] Matsumoto M., Takemori S., Tahara Y. (2020). Lactic Acid Permeation through Deep Eutectic Solvents-Based Polymer Inclusion Membranes. Membranes.

[3] Mancilla-Rico A., de Gyves J., Rodríguez de San Miguel E. (2021). Structural Characterization of the Plasticizers’ Role in Polymer Inclusion Membranes Used for Indium (III) Transport Containing IONQUEST^®^ 801 as Carrier. Membranes.

[4] García-Beleño J., Rodríguez de San Miguel E. (2021). Integration of Response Surface Methodology (RSM) and Principal Component Analysis (PCA) as an Optimization Tool for Polymer Inclusion Membrane Based-Optodes Designed for Hg(II), Cd(II), and Pb(II). Membranes.

